# Poxviral ANKR/F-box Proteins: Substrate Adapters for Ubiquitylation and More

**DOI:** 10.3390/pathogens11080875

**Published:** 2022-08-03

**Authors:** Robert J. Ingham, Farynna Loubich Facundo, Jianing Dong

**Affiliations:** Department of Medical Microbiology and Immunology and Li Ka Shing Institute of Virology, Katz Group Centre for Pharmacy and Health Research, University of Alberta, Edmonton, AB T6G 2E1, Canada; facundo@ualberta.ca (F.L.F.); jianing2@ualberta.ca (J.D.)

**Keywords:** poxvirus, ubiquitin, E3 Ub-ligases, ankyrin repeats, F-box, protein degradation

## Abstract

Poxviruses are double-stranded DNA viruses that infect insects and a variety of vertebrate species. The large genomes of poxviruses contain numerous genes that allow these viruses to successfully establish infection, including those that help evade the host immune response and prevent cell death. Ankyrin-repeat (ANKR)/F-box proteins are almost exclusively found in poxviruses, and they function as substrate adapters for Skp1-Cullin-1-F-box protein (SCF) multi-subunit E3 ubiquitin (Ub)-ligases. In this regard, they use their C-terminal F-box domain to bind Skp1, Cullin-1, and Roc1 to recruit cellular E2 enzymes to facilitate the ubiquitylation, and subsequent proteasomal degradation, of proteins bound to their N-terminal ANKRs. However, these proteins do not just function as substrate adapters as they also have Ub-independent activities. In this review, we examine both Ub-dependent and -independent activities of ANKR/F-box proteins and discuss how poxviruses use these proteins to counteract the host innate immune response, uncoat their genome, replicate, block cell death, and influence transcription. Finally, we consider important outstanding questions that need to be answered in order to better understand the function of this versatile protein family.

## 1. Poxviruses Are Double-Stranded DNA Viruses That Infect a Range of Animals

Poxviruses are brick- or ovoid-shaped double-stranded DNA viruses [1]. The *Poxviridae* family consists of two subfamilies: the *Entomopoxvirinae*, which infect insects, and the *Chordopoxvirinae*, which infect vertebrates. Within the *Chordopoxvirinae*, the 2020 report by the International Committee on the Taxonomy of Viruses (ICTV) recognizes 18 genera, including viruses that infect a wide range of vertebrate species [2]. These include human pathogens such as variola virus (VARV), monkeypox virus (MPXV), and molluscum contagiosum [3,4,5,6], as well as viruses that infect economically important livestock species such as Orf virus (ORFV), sheeppox virus (SPXV), and goatpox virus (GPXV) [7,8,9]. The importance of poxviruses is further highlighted by their use as vaccine vectors for viral and non-viral pathogens [10,11], and their development as oncolytics to treat cancer [12,13]. Thus, much research has focused on understanding how poxviruses interact with the cells and organisms they infect.

The genomes of poxviruses range from ~140 to 300 kb, and they possess, on average, ~200 genes [1,14,15]. The linear genome is closed at both ends by hairpin structures within the inverted terminal repeats. The central region of the genome is highly conserved between poxviruses and contains many of the genes required for the virus to replicate and produce virus particles [14]. The arms are more variable and contain genes that control host range [16,17], prevent cell death [18,19], and help evade the host immune response [20,21,22]. Several of these genes allow these viruses to co-opt or subvert the cellular ubiquitin (Ub)–proteasome system (UPS).

## 2. Poxviruses Co-Opt and Exploit the Host Ub–Proteasome System

Ub is a 76-amino-acid polypeptide that is present in all eukaryotic organisms. It is highly conserved, with only three amino acid differences between human and *S. cerevisiae* Ub [23]. The modification of lysine residues in proteins with Ub, termed ubiquitylation, is best known for targeting proteins to the 26S proteasome or lysosome for degradation [24,25]. However, it also serves non-degradative functions through altering protein function, mediating protein–protein interactions, and controlling the subcellular localization of proteins [26,27].

**Figure 1:**
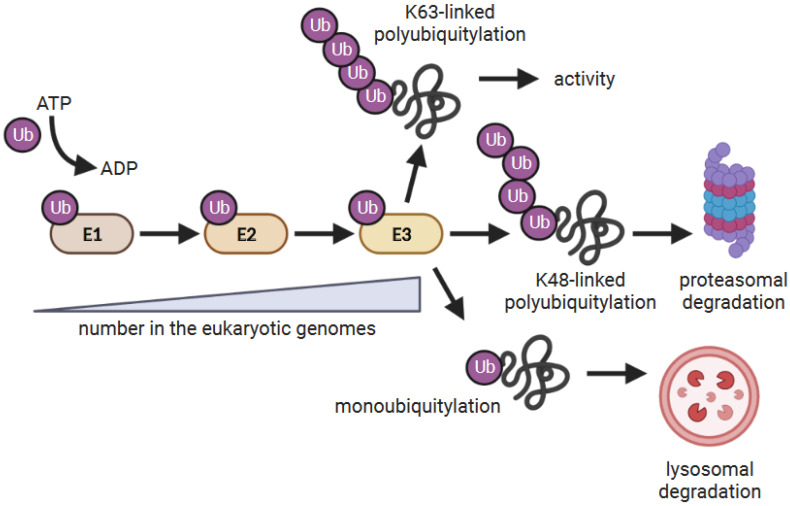
The ubiquitylation reaction. E1 Ub-activating enzymes are charged with Ub and transfer the Ub to E2 Ub-conjugating enzymes. E2 enzymes either directly or indirectly associate with E3 Ub ligases which facilitates the ubiquitylation of substrates. Some E3s have E2 and substrate-binding domains as part of the same polypeptide, while others function as multiple protein complexes where E2 and substrate binding are mediated by different proteins. The consequence of ubiquitylation depends on how the target proteins are modified by Ub [26,27]. Proteins modified with a single Ub (monoubiquitylated) are often targeted to the lysosome for degradation, whereas proteins modified with lysine (K)-48-linked poly Ub chains are targeted to the proteasome for degradation. K63-linked poly Ub chains are often associated with altering protein function.

The ubiquitylation reaction utilizes three enzymes (Figure 1). E1 Ub-activating enzymes activate Ub in an ATP-dependent manner and then transfer this Ub to an E2 Ub-conjugating enzyme. E2s associate with E3 Ub ligases, which facilitates substrate ubiquitylation [28,29]. E3s exist as single proteins or multi-protein complexes. In the latter, E2 and substrate binding are mediated by separate proteins within the complex. Eukaryotes have over 600 E3 Ub-ligases [30] which can be divided into three classes: HECT (homologous to E6-AP C-terminus), RING (really interesting new gene), and RBR (ring-in-between-ring) proteins [28]. Poxviruses require components of the cellular ubiquitylation machinery and the proteasome to replicate [31,32,33,34]. Moreover, they co-opt and exploit this system to modulate the host’s innate and adaptive immune responses [35,36,37,38,39]. Many poxviral proteins are themselves ubiquitylated [31], and it is estimated that Ub comprises ~3% of the total protein in the virion [40]. Some poxviruses even encode Ub-like molecules [41,42,43], but it is unclear if they are involved in Ub transfer.

Poxviruses have also acquired a diverse collection of E3 Ub ligases. These include RING domain-containing E3 Ub ligases, and substrate adapters for multi-subunit cellular RING domain-containing Ub ligases. The former include MARCH (membrane-associated RING-CH) and p28 proteins, and the latter consist of ANKR/F-box (ankyrin repeat/F-box), BTB/Kelch (broad-complex, tramtrack and bric à brac/kelch), and ANKR/BC-box families (reviewed in [44,45,46,47]). Cellular counterparts of ANKR/BC-box and BTB/Kelch proteins are common, whereas ANKR/F-box proteins are extremely rare in eukaryotes and p28 proteins have only been found in poxviruses. Moreover, considerable gene duplication has occurred for ANKR/F-box and BTB-Kelch proteins [44,45,46,47]. In this review, we focus on how the duplication of ANKR/F-box genes has generated proteins with a remarkable array of functions.

## 3. Poxviral ANKR/F-box Proteins

**Figure 2:**
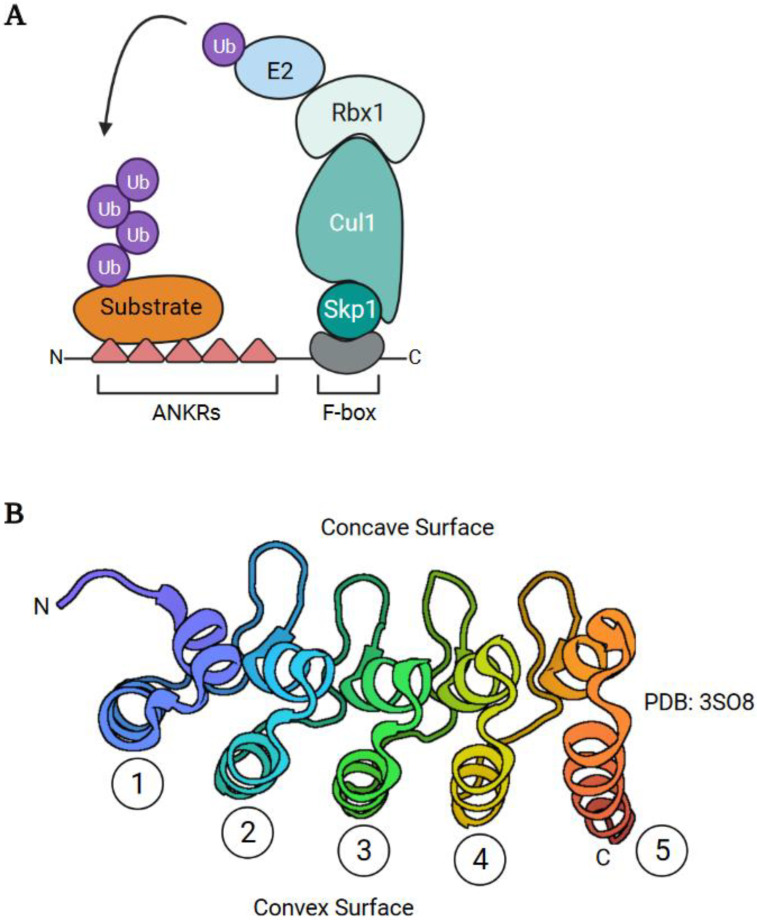
ANKR/F-box substrate adapters. (**A**) Poxviral ANKR/F-box proteins function as substrate adapters using their C-terminal F-box domain to recruit Skp1, Cullin-1, Rbx1, and E2 enzymes to facilitate the ubiquitylation of substrates bound to their ANKRs. As discussed in the text, some poxviral ANKR/F-box proteins also have degradation-independent activities. (**B**) Structure of the 5 ANKRs (PDB:3SO8) of the human ANKRA protein [57]. The independent ANKRs are numbered and the concave and convex surfaces are illustrated.

ANKR/F-box proteins are found in most *Chordopoxvirinae* genera, but a few species such as molluscum contagiosum virus, crocodilepox virus, and red squirrel poxvirus have none and appear to have lost all ANKR proteins [48,49,50]. ANKR/F-box proteins consist of a number of N-terminal ANKRs coupled to a C-terminal F-box domain (Figure 2A). ANKRs are named for a repeated motif identified in the ankyrin protein which possesses 24 repeats [51]. They are found throughout eukaryotes, and present in many prokaryotes, archaea, and viruses [52]. ANKR proteins have a variety of other protein domains, and they regulate a variety of cellular activities including the cell cycle, cytoskeleton, signal transduction, and transcription [53,54,55]. Individual repeats are ~33 amino acids in size and adopt a helix–loop–helix–β–hairpin/loop structure [56]. Arrays of 2 to over 20 of these motifs generate structures with concave and convex surfaces, with the concave surface primarily functioning as a protein–protein interaction interface (Figure 2B) [55,56].

The F-box was first identified in the cell cycle protein, Cyclin F [58], but has since been identified in other proteins throughout eukaryotes [59,60,61,62,63]. Pertinent to this review, some eukaryotic F-box proteins function as substrate adaptors for Skp1-Cullin-1-F-box protein (SCF) multi-subunit E3 Ub ligases. These proteins use their F-box to recruit E2 enzymes via Skp1, Cullin-1, and the RING domain-containing protein, Rbx1 (Figure 2A). This is coupled to a protein–protein interaction (WD repeats, LRR (leucine-rich repeat) domain) to recruit substrates [60,64]. F-box domains consist of three alpha helices and are 40 to 50 amino acids in size [65]. The F-box domains of poxviruses are truncated, consisting of only the first two alpha helices [66,67], and are part of the larger PRANC (pox protein repeats of ankyrin C-terminal) domain (PFAM; PF09372) identified at the C-terminus of poxviral ANKR/F-box proteins. Nonetheless, truncated poxviral F-box domains can interact with Skp1/Cul1 ([67,68,69,70], as examples).

ANKR/F-box proteins are extremely rare in eukaryotes having only been identified in parasitic wasps (*Nasonia* sp.). However, they have been found in some bacterial species including symbionts of *Nasonia* [71,72,73]. Since ANKR/F-box proteins are not found in the *Entomopoxvirinae*, it is unclear how chordopoxviruses could have acquired ANKR/F-box proteins through a direct horizontal gene transfer event from a eukaryotic host. Alternatively, these proteins may have been generated in an ancestral poxvirus species through the recombination of separate ANKR and F-box domains. More recently, Odon et al. have argued that the acquisition of an ANKR/BC-box protein, which have many eukaryotic homologues, may have been the founding event that led to the generation of ANKR/F-box proteins [74].

**Figure 3:**
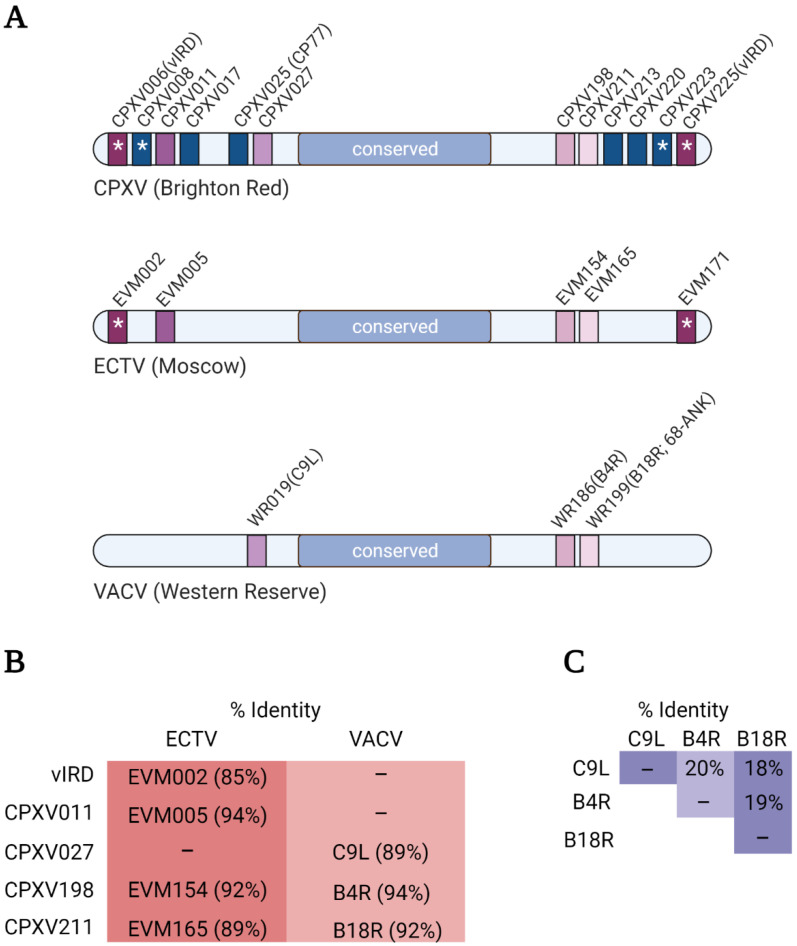
ANKR/F-box genes from select orthopoxviruses. (**A**) Cartoons illustrating the ANKR/F-box genes of CPXV (Brighton Red strain), ECTV (Moscow strain), and VACV (Western Reserve strain). Orthologous genes between viruses have the same colour, and for simplicity, fragmented genes are not shown. (**B**) The overall percent amino acid identity between the indicated CPXV protein and its orthologues in ECTV and VACV was determined using the EMBOSS Needle alignment tool using default settings [81]. (**C**) The overall percent amino acid identity between the three VACV proteins was determined as described in (**B**).

Regardless of how poxviruses have acquired ANKR/F-box proteins, the genes encoding for these proteins have undergone remarkable duplication (Figure 3A). This is best illustrated by the canarypox virus which has 35 reported ANKR/F-box genes [50]. Phylogenetic analyses have revealed that duplications have occurred primarily within genera (or related genera), and orthologous genes within genera show a high degree of sequence identity [48,49,50] (Figure 3B,C). Identical copies of ANKR/F-box genes can sometimes be found in both arms of the genome, and there is also evidence that orthologous ANKR/F-box proteins have similar functions [19,75,76]. However, orthopoxviruses have lost or possess fragmented ANKR/F-box genes. For example, CPXV is thought to be most like the ancestral orthopoxvirus [77], and possesses 12 putative ANKR/F-box proteins, whereas ECTV and VACV possess far fewer intact genes (Figure 3A) [48,49,50]. A correlation between the number of ANKR/F-box proteins and host range has been argued [48,49], and several ANKR/F-box proteins influence host range ([78,79,80] as examples).

In this review, we discuss the roles that select poxviral ANKR/F-box proteins perform during infection (summarized in Table 1). We have chosen examples from multiple genera which highlight the range of functions performed by these proteins. Furthermore, we examine the Ub-dependent and -independent activities of poxviral ANKR/F-box proteins.

## 4. C9—An Antagonist of the Type I Interferon Response

VACV C9L (WR019) encodes for a 75 kDa ANKR/F-box protein with orthologues in other orthopoxviruses. C9L is expressed early during infection, and clues to the protein’s function were revealed by a study examining the ability of VACV Western Reserve (WR) deletion mutants to replicate in interferon-β (IFN-β) pre-treated A549 cells [37]. While VACV WR was able replicate in pre-treated cells, a virus with a large deletion in the left arm of the genome was found to be sensitive to this pre-treatment. Further investigation identified C9L as the gene conferring IFN-β resistance, and the deletion of C9L from VACV WR resulted in a virus that was impaired in its ability to transcribe intermediate and late genes, and uncoat and replicate its genome when treated with IFN-β [37]. In addition, co-immunoprecipitation and mass spectrometry experiments identified Cul1 and Skp1 in association with C9, which suggests that the protein functions as a substrate adapter to facilitate the ubiquitylation of cellular or viral proteins [37].

To determine the cellular function of C9, a follow-up mass spectrometry study identified proteins that preferentially associated with a C9 protein lacking the F-box domain (C9 ΔF-box) compared to intact C9 [38]. The rationale for this approach was that, by uncoupling the ANKRs from the F-box-associated ubiquitylation machinery, this would trap substrates that transiently interact with C9 and/or are quickly targeted for degradation. This approach yielded three members of the IFN-induced proteins with tetratricopeptide repeats (IFITs) family of proteins—IFIT1, IFIT2, and IFIT3—which were specifically enriched in the immunoprecipitates of the C9 ΔF-box protein.

IFITs are part of the myriad of interferon-stimulated genes (ISGs) that are transcribed by cells in response to type I and III IFNs. These ISGs include proteins that inhibit different steps of the virus life cycle including entry, viral gene transcription and translation, genome replication, and virion assembly and egress [82]. IFIT mRNAs are rapidly induced in response to type I IFN signalling [83], and they recognize and bind features specific to the 5′ cap of viral RNAs including triphosphorylation [84] and 2′-*O*-unmethylation [85] in order to prevent the translation of viral RNAs. VACV can overcome the latter by methylating viral RNAs at the 2′-*O* position using the ribose methyltransferase activity encoded by the J3 protein [86].

**Figure 4:**
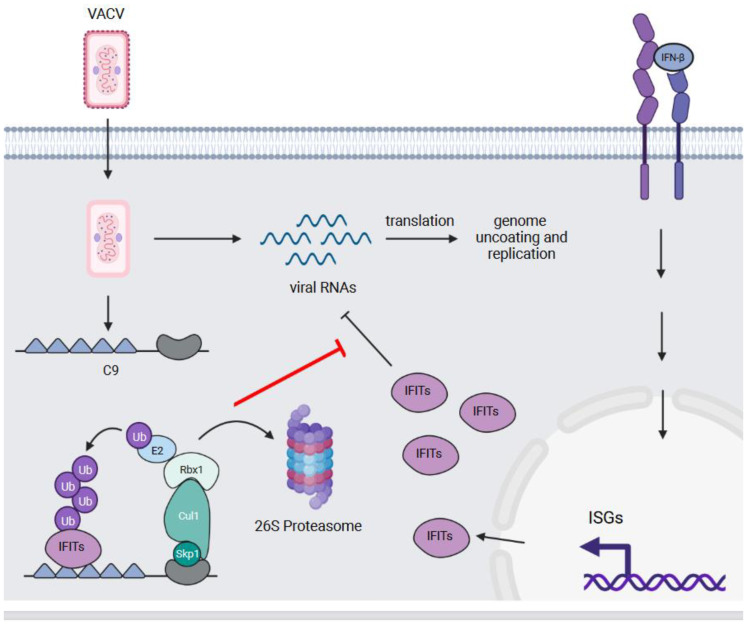
C9—an antagonist of the type I interferon response. IFITs are ISGs that are induced in response to the stimulation of cells with IFN-β and other type I and III interferons. These proteins bind triphosphorylated and 2′ *O*-unmethylated viral RNAs to prevent their translation. The C9 protein of VACV binds and ubiquitylates multiple IFITs, which results in their proteasomal degradation. This counteracts the anti-viral activity of IFITs and allows VACV to uncoat and replicate its genome.

The ubiquitylation and degradation of IFITs by C9 would represent an additional mechanism to thwart the antiviral effects of IFIT proteins. In support of this notion, Liu et al. demonstrate that C9 ubiquitylates IFITs and targets them for proteasomal degradation [38] (Figure 4). Moreover, the C9 ΔF-box virus was comparable to the complete C9-deleted virus with respect to blocking viral gene expression in response to IFN-β treatment [38]. This implies that C9 needs to target IFITs for degradation, not merely sequester them, in order to overcome the antiviral activity of these proteins [38]. Thus, C9, in conjunction with J3, provides VACV, an elegant two-pronged approach to counteract the antiviral activity of the IFIT proteins.

## 5. vIRD—An Inhibitor of Necroptotic Cell Death and Inflammatory Signalling

**Figure 5:**
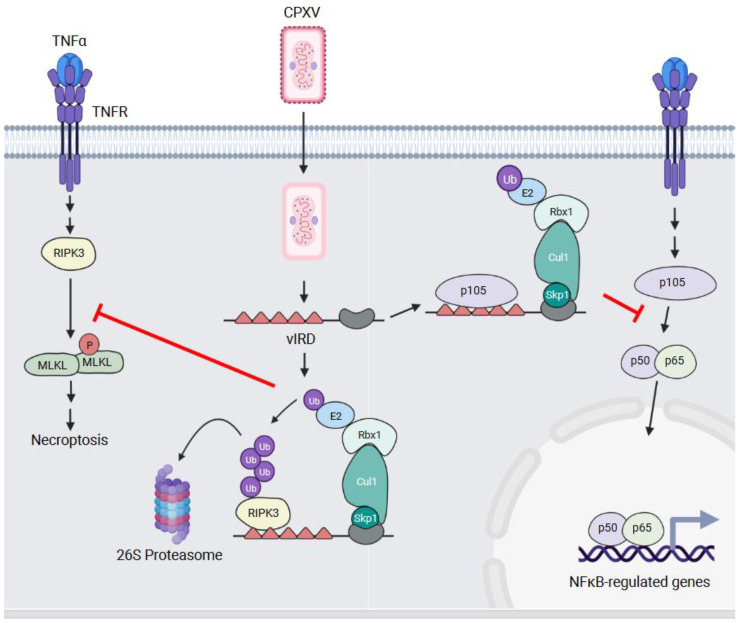
vIRD—a regulator of necroptotic and inflammatory signalling. TNFα binding to the TNFR initiates signalling events that lead to the activation of the RIPK3 serine/threonine kinase. RIPK3 phosphorylates the MLKL protein which results in MLKL multimerization, membrane permeabilization, and the induction of necroptosis. In CPXV-infected cells, vIRD blocks necroptosis by preventing MLKL phosphorylation through binding, ubiquitylating, and targeting RIPK3 to the proteasome for degradation. vIRD also binds the p105 NFKB1 protein, but it does not promote p105 NFKB1 degradation. Rather, this interaction appears to block the processing of p105 NFKB1 into the p50 subunit. This prevents p50-containing NF-κB dimers from translocating to the nucleus and promoting the transcription of NF-κB-regulated genes in response to TNFR signalling.

Amongst orthopoxviruses, CPXV possesses the most ANKR/F-box proteins within its genome (Figure 3A). Viral inducer of RIPK3 degradation (vIRD), also known as CPXV006, is one such protein that functions both as an inhibitor of necroptosis and of the NF-κB transcription factor (Figure 5).

Necroptosis is a form of regulated cell death and is induced in some virus-infected cells to prevent virus replication [87]. This is an inflammatory process and results in the release of danger-associated molecular patterns (DAMPS) from the infected cells. Signals that initiate necroptosis converge on receptor-interacting protein kinase 3 (RIPK3) which is a serine/threonine kinase whose activation leads to the phosphorylation and oligomerization of mixed lineage kinase domain-like (MLKL) proteins. Oligomerized MLKL forms pores in the membrane that allows for the release of cellular DAMPs [88].

VACV sensitizes infected cells to tumour necrosis factor (TNF)-mediated necroptosis [89], and Liu and colleagues investigated whether this was true of other poxviruses [19]. They found that while MYXV infection sensitized cells to necroptosis, CPXV infection did not. This failure to induce necroptosis in CPXV-infected cells was associated with ubiquitylation and the proteasomal degradation of RIPK3. This suggests that CPXV had a protein(s) that inhibited necroptosis, either indirectly or directly, by targeting RIPK3 for proteasomal degradation. To elucidate the responsible gene(s), the authors examined the CPXV genome for genes present in CPXV that were deleted or fragmented in VACV. They then performed an siRNA screen to examine whether knocking down any of these genes sensitized CPXV-infected cells to necroptosis. This screen identified vIRD as the responsible gene, and not surprisingly, this gene is fragmented in orthopoxviruses that cannot block necroptosis.

The deletion of vIRD, and apparently the identical CPXV225 [90], from CPXV was sufficient to induce RIPK3 degradation and sensitize infected cells to necroptosis [19]. The first five ANKRs of vIRD are necessary for interacting with the RIP homotypic interaction motif (RHIM) domain of RIPK3. The deletion of vIRD also attenuated CPXV virulence in a mice [19,90], and the addition of vIRD to a VACV strain with a fragmented gene increased the sensitivity of infected cells to necroptotic cell death and enhanced pathogenesis in a mouse model [19].

vIRD and its orthologues are not just inhibitors of necroptosis (Figure 5). A yeast two-hybrid screen using the VARV vIRD orthologue, G1R, identified NF-κB1 p105 as an interacting protein [76]. NF-κB transcription factors consist of dimers of the p50, p52, RelA (p65), c-Rel, and RelB proteins [91]. In unstimulated cells, NF-κB dimers are sequestered in the cytosol through binding the inhibitor of κB (IκB) proteins. In response to a variety of stimuli, the IκB proteins are phosphorylated by the IκB kinase (IKK) complex which targets the IκB proteins for Ub-mediated degradation. This enables dimers to translocate to the nucleus where they drive the transcription of genes associated with inflammation, proliferation, and survival [91].

The p50 protein is generated by the cleavage of the NF-κB1 p105 precursor protein. Mohamed et al. demonstrated that NF-κB1 p105 interacts with multiple vIRD orthologues, and this interaction was mediated by the ANKR repeats of vIRD and NF-κB1 p105 [76]. Ectopic vIRD expression stabilized NF-κB1 p105 levels, prevented processing into the p50 isoform, and inhibited TNF-α-induced NF-κB luciferase activity [76]. This was argued to be an Ub-independent process where vIRD binds to NF-κB1 p105 to prevent its phosphorylation by IKKs and processing into the p50 form [90] (Figure 5).

The effect of vIRD on NF-κB signalling was further examined using the CPXV ΔvIRD virus. The infection of cells with this virus resulted in the loss of detectable p105 proteins, the nuclear accumulation of the NF-κB p50/p65 subunits, and the elevated secretion of NF-κB-regulated pro-inflammatory cytokines [90]. Thus, vIRD is a multifunctional protein that plays several roles in helping poxviruses infect cells.

## 6. M-T5—An Activator of the Akt Serine/Threonine Kinase

**Figure 6:**
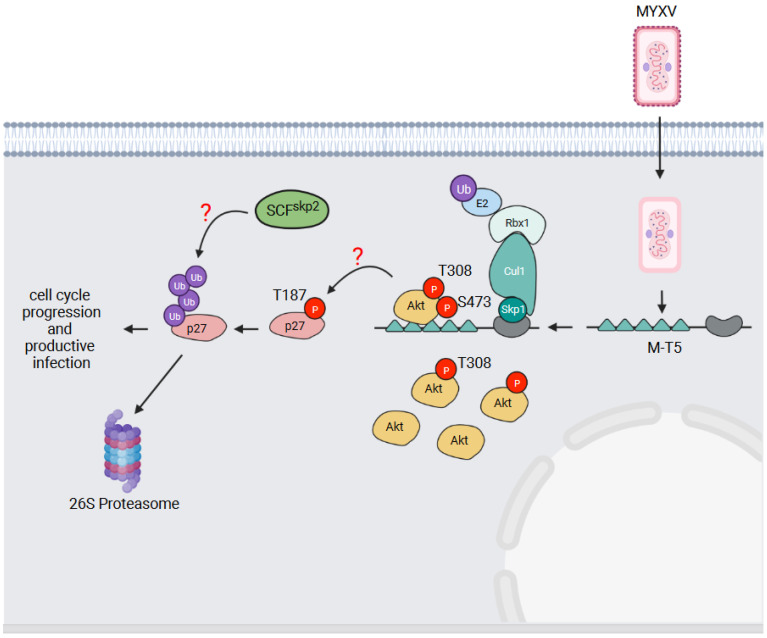
M-T5—an activator of the Akt serine/threonine kinase. The MYXV M-T5 protein binds the cellular Akt serine/threonine kinase when phosphorylated on threonine (T) 308. This promotes the further phosphorylation of Akt on serine (S) 473 to fully activate the kinase. The activation of Akt is proposed to lead to the phosphorylation of the cyclin-dependent kinase inhibitor p27^kip1^ on T187. The phosphorylation of p27^kip1^ on this site is then recognized by the cellular SCF^skp2^ E3 Ub ligase, resulting in the Ub-mediated degradation of p27^kip1^. This overcomes the cell cycle block and allows MYXV infection to proceed.

Myxoma virus (MYXV) causes lethal disease (myxomatosis) in European rabbits (old world), but not rabbits from the Americas (new world) [92]. MYXV has five ANKR/F-box genes: M148R, M149R, M150R and two copies of the M-T5 gene within each of the inverted terminal repeats of the MYXV genome. The deletion of both copies of M-T5 severely attenuates MYXV in rabbits, and ΔM-T5 MYXV is unable to spread from the site of infection [80]. M-T5 has been further shown to influence the host range of MYXV through preventing cell cycle arrest and consequent apoptotic and autophagic cell death [80,93]. Johnston et al. have demonstrated that cell lines permissive to MYXV infection are able to overcome the G_0_/G_1_ block in the cell cycle in a M-T5-dependent manner [93]. In cells infected with ΔM-T5 MYXV, decreased ubiquitylation and increased protein levels of the cyclin-dependent kinase inhibitor p27^kip1^ have been observed [93]. In addition, the decreased phosphorylation of p27^kip1^ on threonine 187 in cells infected with ΔM-T5 MYXV, which is known to target p27^kip1^ for degradation by the cellular SCF^skp2^ E3 Ub-ligase complex, has also been observed [94]. Since no evidence demonstrating that p27^kip1^ associates with M-T5 has been presented to our knowledge, M-T5 may not directly ubiquitylate p27^kip1^. Instead, the function of MT-5 may be to promote p27^kip1^ phosphorylation for recognition by another E3 Ub-ligase, perhaps SCF^skp2^ (Figure 6). How M-T5 might facilitate this phosphorylation has been revealed by studies showing that M-T5 binds and activates the Akt serine/threonine kinase [70,95,96].

In addition to a kinase domain, Akt possesses an N-terminal pleckstrin homology (PH) domain which binds phosphorylated lipid substrates of phosphatidyl inositol 3-kinase (PI3K) [97]. Lipid-binding activates Akt, but in order to become fully activated, Akt also requires phosphorylation on threonine 308 and serine 473 [97,98]. Threonine 308 phosphorylation is mediated by phosphoinositide-dependent kinase-1 (PDK1) [99], and serine 473 has been reported to be phosphorylated by mammalian target of rapamycin complex 2 (mTORC2) [100]; although, whether mTORC2 actually phosphorylates this site has recently been questioned [101]. Through the phosphorylation of several protein substrates, Akt regulates a variety of cellular activities including apoptosis, cell cycle progression, metabolism, and cell growth [97].

The ability of MYXV, and the corresponding M-T5 deletion virus, to productively infect different human cell lines has been found to correlate with the phosphorylation status of Akt [95]. Cell lines with high levels of Akt phosphorylation (type I cells) could be productively infected by both viruses. Cell lines with low levels of Akt phosphorylation (type II cells) could be productively infected with MYXV, but not ΔM-T5 MYXV. Those with no observable Akt activation (type III cells) could not be productively infected by either virus [95]. In type II cell lines, MYXV infection increased Akt phosphorylation in a M-T5-dependent manner, and importantly, without affecting Akt protein levels [95,102]. M-T5 interacts with Akt via its first two ANKRs [70,95], and this interaction is dependent on Akt phosphorylation at threonine 308 [102]. The recruitment of Akt to M-T5 leads to the further phosphorylation of Akt on serine 473 which fully activates the kinase [102] (Figure 6). Thus, despite M-T5 being able to simultaneously recruit both Akt and Skp1 [70], it does not appear to target Akt for Ub-mediated degradation. Rather, M-T5 has been argued to activate Akt in an analogous manner to the cellular PIKE-A protein which also binds and activates Akt [96]. The simplest working model for how M-T5 blocks cell cycle arrest is illustrated in Figure 6. M-T5 binds and fully activates Akt which then phosphorylates p27^kip1^ on threonine 187 [103]. Phosphorylated p27^kip1^ is then recognized by the cellular SCF^skp2^ E3 Ub ligase complex [94], which ubiquitylates p27^kip1^ and targets the protein for proteasomal degradation. It has even been suggested that M-T5 might serve as scaffold to co-localize Akt and p27^kip1^ to better facilitate p27^kip1^ phosphorylation [70], but this has not been experimentally proven.

## 7. ORFV ANKR/F-box Proteins—Sequestering FIH to Facilitate HIF Signalling

**Figure 7:**
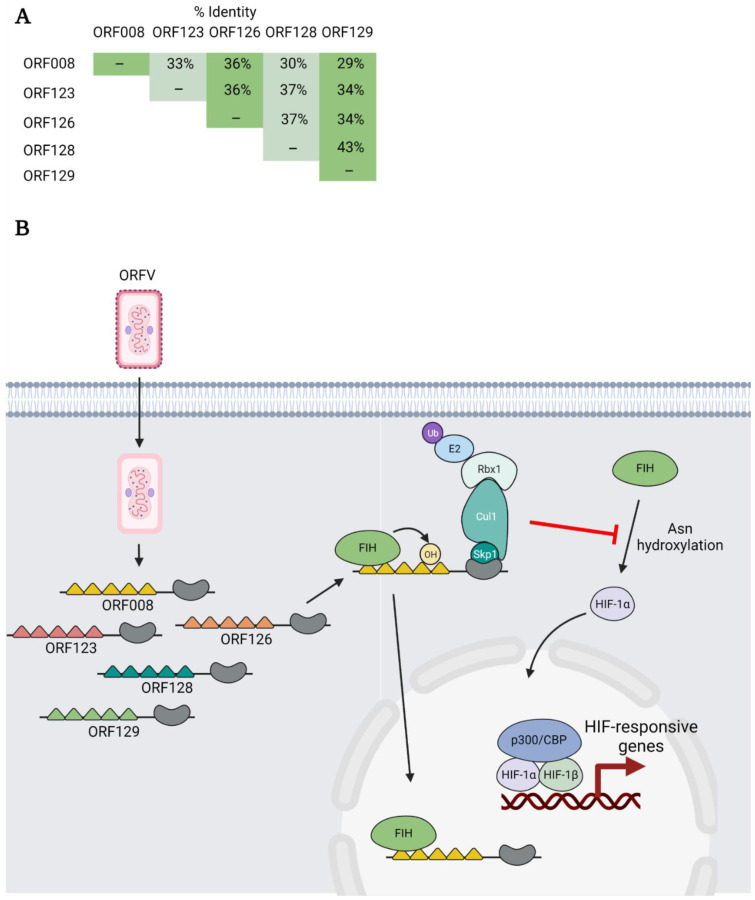
ORFV ANKR/F-box proteins—sequestering FIH to facilitate HIF signalling. (**A**) The overall percent amino acid identity between each of the ORFV ANKR/F-box proteins was determined using the EMBOSS Needle alignment tool using default settings. (**B**) Multiple ORFV ANKR/F-box proteins bind and are hydroxylated on asparagine (Asn) residues by the cellular enzyme factor-inhibiting HIF (FIH). Note: the ability of the different proteins to bind and be hydroxylated by FIH varies. The ORFV ANKR/F-box proteins are thought to activate HIF transcriptional activity by binding and sequestering FIH, perhaps in the nucleus. This prevents the asparaginyl hydroxylation of HIF-1α which would otherwise impair HIF-1α transcriptional activity by blocking the recruitment of the transcriptional coactivators p300/CBP.

ORFV is a member of the parapoxvirus genus and primarily infects sheep and goats, but humans can be zoonotically infected through contact with infected animals [9]. ORFV encodes five ANKR/F-box proteins: ORF008, ORF123, ORF126, ORF128, and ORF129, and each can bind to Skp1 and Cul1 [68]. These proteins have overall amino acid identities of 29–43%, as determined using the EMBOSS Needle alignment tool [81] (Figure 7A), and intriguing data from Chen and colleagues demonstrate that these proteins regulate hypoxic signalling during infection [104] (Figure 7B).

Hypoxia-inducible factor (HIF) is a dimeric transcription factor consisting of α and β subunits. Under conditions of low oxygen (hypoxia), HIF promotes the transcription of genes that regulate metabolism, angiogenesis, cell cycle, inflammation and other processes [105]. Under normal (normoxic) conditions, HIF-1α is hydroxylated on proline residues by HIF prolyl hydroxylases (PHDs). This leads to the recognition of HIF-1α by the VHL multi-subunit E3 Ub ligase, and the targeting of the protein for proteasomal degradation. HIF-1α is also regulated by the hydroxylation of asparagine residues by factor-inhibiting HIF (FIH) [105]. Rather than targeting HIF-1α for degradation, asparagine hydroxylation decreases HIF transcriptional activity by impairing association with the transcriptional coactivators p300/CBP [106]. Thus, a change in cellular oxygen levels creates a sensitive system for the regulation of HIF-responsive genes.

In addition to HIF-1α, FIH also associates with and hydroxylates several cellular ANKR domain-containing proteins [107]. This led Chen and colleagues to examine whether this was true of poxviral ANKR/F-box proteins. They demonstrated that each of the five ANKR/F-box proteins of ORFV, to varying degrees, could interact with FIH. For ORF008, this interaction was shown to be mediated by the first and fourth ANKRs. In addition, several ORFV ANKR/F-box proteins could be hydroxylated by FIH in vitro, but the significance of this hydroxylation is unknown (Figure 7B).

Further experiments revealed that FIH levels were not decreased during ORFV infection or when ORFV ANKR/F-box proteins were ectopically expressed in cells [104]. Instead, the authors presented evidence that ORFV ANKR/F-box proteins activate HIF signalling by sequestering FIH, perhaps in the nucleus, to prevent FIH from hydroxylating HIF-1α and impair its transcriptional activity [104] (Figure 7B). Intriguingly, the VACV C16 protein also promotes HIF signalling using a distinct mechanism. C16 binds PHD2 and stabilizes HIF-α by preventing its proline hydroxylation. Thus, poxviruses have multiple strategies to increase HIF transcriptional activity. Why these viruses target the HIF pathway is currently unknown; however, it has been postulated that it may provide additional resources to support virus replication [104].

## 8. Summary and Future Perspectives

Poxviruses have a remarkable number of ANKR/F-box proteins, and they use these proteins in a variety of ways to allow these viruses to productively infect cells. They enable poxviruses to uncoat and replicate their genomes, inhibit the host innate immune response, and impair cell death. ANKR/F-box proteins function as classical substrate adapters for multi-subunit E3 Ub ligases and target proteins for Ub-mediated proteasomal degradation. However, they also have Ub-independent activities illustrating the versatility of these proteins. While we have highlighted some of the better characterized poxviral ANKR/F-box proteins in this review, there are still many important questions and gaps in our knowledge with respect to how these proteins function.

The first gap is that interacting partners and/or substrates still need to be identified for many poxviral ANKR/F-box proteins. For example, modified VACV Ankara (MVA) is a severely attenuated strain that is generated by serial passages in chick embryo fibroblasts [108]. MVA possesses a single ANKR/F-box gene, 68 kDa ankyrin-like protein (68k-ank), which influences the MVA host range and is required for MVA to uncoat and replicate its genome [109,110]. How 68k-ank regulates these processes is not known. Intriguingly, at least some of these activities are independent of the F-box domain [109]. Likewise, VACV B4R and its ECTV orthologue, EVM154, mediate virus spread via an unknown mechanism [75]. Thus, previously utilized methodologies to identify ANKR/F-box-interacting proteins, such as yeast two-hybrid and mass spectrometry, will be critical to determine the details for how other ANKR/F-box proteins function.

As mentioned, at least some 68k-ank activities are F-box independent [109], and other poxviral ANKR/F-box proteins also function in this manner. CPV77 of CPXV mediates host range in a F-box domain-independent manner, but requires the F-box to interfere with NF-κB signalling [111]. As well, the ECTV ANKR/F-box protein, EVM005, also requires the F-box domain to inhibit NF-κB signalling, but a truncated EVM005 lacking the F-box domain is sufficient to rescue virulence associated with EVM005 deletion [112]. The vIRD inhibition of NF-κB signalling, the M-T5 activation of Akt, and the ORFV ANKR/F-box protein inhibition of FIH discussed in this review are also likely F-box-independent activities, but this still needs to be formally investigated and the non-degradative role of Ub ruled out. The fact that some poxviral ANKR/F-box proteins can function independent of their F-box domain raises the possibility that perhaps some fragmented ANKR/F-box proteins may retain functions associated with their intact orthologues [50].

Another unresolved question is: how much conservation in function is there between orthologues? The ability to block necroptosis and NF-κB signalling is conserved amongst vIRD orthologues [19,76], and both VACV B4R and ECTV EVM154 regulate virus spread [75]. Given the high degree of amino acid identity between putative orthologues within genera [48,49,50] (Figure 3B), it is likely that many of these functions are conserved. A related question is: how much redundancy exists between ANKR/F-box proteins within individual poxviruses? Some poxviruses have identical ANKR/F-box at both ends of their genomes, and the ability to sequester FIH appears to be conserved amongst ORFV ANKR/F-box proteins [104]. Likewise, multiple ECTV ANKR/F-box proteins can inhibit the nuclear translocation of NF-κB [113]. With the large number of ANKR/F-box proteins present in the genomes of many poxviruses, it is likely that additional functional redundancies exist. Intriguingly, functional redundancy with respect to ANKR/F-box protein function even extends to unrelated proteins. Liu et al. demonstrated that phenotypes associated 68k-ank deletion from MVA could be rescued by two unrelated VACV proteins: C5, a BTB/Kelch E3 Ub ligase, or M2, a member of the poxvirus immune evasion superfamily [110]. In addition, the CPXV ANKR/F-box protein, CP77, has rescued host range defects in VACV associated with the deletion of the K1L gene, which encodes for ANKR protein without an F-box, or the C7L gene, which encodes for a protein with no identifiable domains [78,79]. Thus, redundancy with respect to poxviral ANKR/F-box protein function is complex and illustrates the importance of back-up mechanisms for critical steps in the infection cycle of these viruses.

In conclusion, the acquisition of ANKR/F-box proteins by poxviruses, and their subsequent expansion, have equipped these viruses with versatile tools to establish infection. It will be interesting to see what novel cellular activities and modes of action future research will reveal for these proteins.

## Figures and Tables

**Table 1:** Summary of ANKR/F-box genes discussed in this review.

E3 Ligase(s)	Poxvirus	Cellular Target Recognized	Target Degradation (Yes/No)	Role during Infection
C9	VACV	IFITs	Yes	Block IFIT anti-viral activity
vIRD	CPXV	RIPK3	Yes	Prevent necroptosis-associated inflammation
		NF-κB1 p105	No	Prevent expression of NF-κB-associated inflammatory genes
M-T5	MYXV	Akt	No	Allow for cell cycle progression and productive infection
ORFs 008, 123, 126, 128, and 129	ORFV	FIH	No	Promote expression of HIF-responsive genes to support viral replication?

## Data Availability

Not applicable.

## References

[1] Moss B., Fields B.N., Knipe D.M., Howley P.M. (2013). Fields virology. Poxviridae.

[2] ICTV Current ICTV Taxonomy Release. https://ictv.global/taxonomy/.

[3] Wehrle P.F. (1980). A Reality in Our Time—Certification of the Global Eradication of Smallpox. J. Infect. Dis..

[4] Yinka-Ogunleye A., Aruna O., Dalhat M., Ogoina D., McCollum A., Disu Y., Mamadu I., Akinpelu A., Ahmad A., Burga J. (2019). Outbreak of human monkeypox in Nigeria in 2017–2018: A clinical and epidemiological report. Lancet Infect. Dis..

[5] Doshi R.H., Guagliardo S.A.J., Doty J.B., Babeaux A.D., Matheny A., Burgado J., Townsend M.B., Morgan C., Satheshkumar P.S., Ndakala N. (2019). Epidemiologic and Ecologic Investigations of Monkeypox, Likouala Department, Republic of the Congo, 2017. Emerg. Infect. Dis..

[6] Romero R.M., Navarrete-Dechent C., Downey C. (2019). Molluscum contagiosum: An update and review of new perspectives in etiology, diagnosis, and treatment. Clin. Cosmet. Investig. Dermatol..

[7] Tuppurainen E.S.M., Venter E.H., Shisler J.L., Gari G., Mekonnen G.A., Juleff N., Lyons N.A., De Clercq K., Upton C., Bowden T.R. (2017). Review: Capripoxvirus Diseases: Current Status and Opportunities for Control. Transbound. Emerg. Dis..

[8] Matos A.C.D., Rehfeld I.S., Guedes M., Lobato Z.I.P. (2018). Bovine Vaccinia: Insights into the Disease in Cattle. Viruses.

[9] Bukar A.M., Jesse F.F.A., Abdullah C.A.C., Noordin M.M., Lawan Z., Mangga H.K., Balakrishnan K.N., Azmi M.-L.M. (2021). Immunomodulatory Strategies for Parapoxvirus: Current Status and Future Approaches for the Development of Vaccines against Orf Virus Infection. Vaccines.

[10] Nagata L.P., Irwin C.R., Hu W.-G., Evans D.H. (2018). Vaccinia-based vaccines to biothreat and emerging viruses. Biotechnol. Genet. Eng. Rev..

[11] Liu F., Zhang H., Liu W. (2019). Construction of recombinant capripoxviruses as vaccine vectors for delivering foreign antigens: Methodology and application. Comp. Immunol. Microbiol. Infect. Dis..

[12] Torres-Domínguez L.E., McFadden G. (2019). Poxvirus oncolytic virotherapy. Expert Opin. Biol. Ther..

[13] Guo Z.S., Lu B., Guo Z., Giehl E., Feist M., Dai E., Liu W., Storkus W.J., He Y., Liu Z. (2019). Vaccinia virus-mediated cancer immunotherapy: Cancer vaccines and oncolytics. J. Immunother. Cancer.

[14] Lefkowitz E., Wang C., Upton C. (2006). Poxviruses: Past, present and future. Virus Res..

[15] Gubser C., Hue S., Kellam P., Smith G.L. (2004). Poxvirus genomes: A phylogenetic analysis. J. Gen. Virol..

[16] Reynolds M.G., Guagliardo S.A.J., Nakazawa Y.J., Doty J.B., Mauldin M.R. (2018). Understanding orthopoxvirus host range and evolution: From the enigmatic to the usual suspects. Curr. Opin. Virol..

[17] Haller S.L., Peng C., McFadden G., Rothenburg S. (2014). Poxviruses and the evolution of host range and virulence. Infect. Genet. Evol..

[18] Suraweera C.D., Hinds M.G., Kvansakul M. (2020). Poxviral Strategies to Overcome Host Cell Apoptosis. Pathogens.

[19] Liu Z., Nailwal H., Rector J., Rahman M.M., Sam R., McFadden G., Chan F.K. (2021). A class of viral inducer of degradation of the necroptosis adaptor RIPK3 regulates virus-induced inflammation. Immunity.

[20] Lawler C., Brady G. (2020). Poxviral Targeting of Interferon Regulatory Factor Activation. Viruses.

[21] Yu H., Bruneau R., Brennan G., Rothenburg S. (2021). Battle Royale: Innate Recognition of Poxviruses and Viral Immune Evasion. Biomedicines.

[22] Albarnaz J.D., Torres A.A., Smith G.L. (2018). Modulating Vaccinia Virus Immunomodulators to Improve Immunological Memory. Viruses.

[23] Özkaynak E., Finley D., Varshavsky A. (1984). The yeast ubiquitin gene: Head-to-tail repeats encoding a polyubiquitin precursor protein. Nature.

[24] Hershko A., Ciechanover A. (1998). The ubiquitin system. Annu. Rev. Biochem..

[25] Saeki Y. (2017). Ubiquitin recognition by the proteasome. J. Biochem..

[26] Komander D., Rape M. (2012). The Ubiquitin Code. Annu. Rev. Biochem..

[27] Swatek K.N., Komander D. (2016). Ubiquitin modifications. Cell Res..

[28] Zheng N., Shabek N. (2017). Ubiquitin Ligases: Structure, Function, and Regulation. Annu. Rev. Biochem..

[29] Pickart C.M., Eddins M.J. (2004). Ubiquitin: Structures, functions, mechanisms. Biochim. Biophys. Acta.

[30] George A.J., Hoffiz Y.C., Charles A.J., Zhu Y., Mabb A.M. (2018). A Comprehensive Atlas of E3 Ubiquitin Ligase Mutations in Neurological Disorders. Front. Genet..

[31] Grossegesse M., Doellinger J., Fritsch A., Laue M., Piesker J., Schaade L., Nitsche A. (2018). Global ubiquitination analysis reveals extensive modification and proteasomal degradation of cowpox virus proteins, but preservation of viral cores. Sci. Rep..

[32] Teale A., Campbell S., Van Buuren N., Magee W.C., Watmough K., Couturier B., Shipclark R., Barry M. (2009). Orthopoxviruses Require a Functional Ubiquitin-Proteasome System for Productive Replication. J. Virol..

[33] Mercer J., Snijder B., Sacher R., Burkard C., Bleck C.K.E., Stahlberg H., Pelkmans L., Helenius A. (2012). RNAi Screening Reveals Proteasome- and Cullin3-Dependent Stages in Vaccinia Virus Infection. Cell Rep..

[34] Satheshkumar P.S., Anton L.C., Sanz P., Moss B. (2009). Inhibition of the Ubiquitin-Proteasome System Prevents Vaccinia Virus DNA Replication and Expression of Intermediate and Late Genes. J. Virol..

[35] Froggatt G.C., Smith G.L., Beard P. (2007). Vaccinia virus gene F3L encodes an intracellular protein that affects the innate immune response. J. Gen. Virol..

[36] Pallett M.A., Ren H., Zhang R.-Y., Scutts S.R., Gonzalez L., Zhu Z., Maluquer de Motes C., Smith G.L. (2019). Vaccinia Virus BBK E3 Ligase Adaptor A55 Targets Importin-Dependent NF-κB Activation and Inhibits CD8^+^ T-Cell Memory. J. Virol..

[37] Liu R., Moss B. (2018). Vaccinia Virus C9 Ankyrin Repeat/F-Box Protein Is a Newly Identified Antagonist of the Type I Interferon-Induced Antiviral State. J. Virol..

[38] Liu R., Olano L.R., Mirzakhanyan Y., Gershon P.D., Moss B. (2019). Vaccinia Virus Ankyrin-Repeat/F-Box Protein Targets Interferon-Induced IFITs for Proteasomal Degradation. Cell Rep..

[39] de Miranda M.P., Reading P., Tscharke D., Murphy B.J., Smith G.L. (2003). The vaccinia virus kelch-like protein C2L affects calcium-independent adhesion to the extracellular matrix and inflammation in a murine intradermal model. J. Gen. Virol..

[40] Chung C.-S., Chen C.-H., Ho M.-Y., Huang C.-Y., Liao C.-L., Chang W. (2006). Vaccinia Virus Proteome: Identification of Proteins in Vaccinia Virus Intracellular Mature Virion Particles. J. Virol..

[41] Afonso C.L., Tulman E.R., Lu Z., Oma E., Kutish G.F., Rock D.L. (1999). The Genome of *Melanoplus sanguinipes* Entomopoxvirus. J. Virol..

[42] Bawden A.L., Glassberg K.J., Diggans J., Shaw R., Farmerie W., Moyer R.W. (2000). Complete Genomic Sequence of the Amsacta moorei Entomopoxvirus: Analysis and Comparison with Other Poxviruses. Virology.

[43] Tulman E.R., Afonso C.L., Lu Z., Zsak L., Kutish G.F., Rock D.L. (2004). The Genome of Canarypox Virus. J. Virol..

[44] Barry M., Van Buuren N., Burles K., Mottet K., Wang Q., Teale A. (2010). Poxvirus Exploitation of the Ubiquitin-Proteasome System. Viruses.

[45] Zhang L., Villa N.Y., McFadden G. (2009). Interplay between poxviruses and the cellular ubiquitin/ubiquitin-like pathways. FEBS Lett..

[46] Cui H., Zhang Y., Zhang L. (2021). Progress on Poxvirus E3 Ubiquitin Ligases and Adaptor Proteins. Front. Immunol..

[47] Lant S., de Motes C.M. (2021). Poxvirus Interactions with the Host Ubiquitin System. Pathogens.

[48] Herbert M.H., Squire C.J., Mercer A.A. (2015). Poxviral Ankyrin Proteins. Viruses.

[49] Bratke K.A., McLysaght A., Rothenburg S. (2012). A survey of host range genes in poxvirus genomes. Infect. Genet. Evol..

[50] Sonnberg S., Fleming S.B., Mercer A. (2011). Phylogenetic analysis of the large family of poxvirus ankyrin-repeat proteins reveals orthologue groups within and across chordopoxvirus genera. J. Gen. Virol..

[51] Lux S.E., John K.M., Bennett V. (1990). Analysis of cDNA for human erythrocyte ankyrin indicates a repeated structure with homology to tissue-differentiation and cell-cycle control proteins. Nature.

[52] Al-Khodor S., Price C.T., Kalia A., Abu Kwaik Y. (2010). Functional diversity of ankyrin repeats in microbial proteins. Trends Microbiol..

[53] Mosavi L.K., Cammett T.J., Desrosiers D.C., Peng Z.-Y. (2004). The ankyrin repeat as molecular architecture for protein recognition. Protein Sci..

[54] Sedgwick S.G., Smerdon S.J. (1999). The ankyrin repeat: A diversity of interactions on a common structural framework. Trends Biochem. Sci..

[55] Islam Z., Nagampalli R.S.K., Fatima M.T., Ashraf G.M. (2018). New paradigm in ankyrin repeats: Beyond protein-protein interaction module. Int. J. Biol. Macromol..

[56] Kane E.I., Spratt D.E. (2021). Structural Insights into Ankyrin Repeat-Containing Proteins and Their Influence in Ubiquitylation. Int. J. Mol. Sci..

[57] Xu C., Jin J., Bian C., Lam R., Tian R., Weist R., You L., Nie J., Bochkarev A., Tempel W. (2012). Sequence-Specific Recognition of a PxLPxI/L Motif by an Ankyrin Repeat Tumbler Lock. Sci. Signal..

[58] Bai C., Sen P., Hofmann K., Ma L., Goebl M., Harper J., Elledge S.J. (1996). SKP1 Connects Cell Cycle Regulators to the Ubiquitin Proteolysis Machinery through a Novel Motif, the F-Box. Cell.

[59] Lechner E., Achard P., Vansiri A., Potuschak T., Genschik P. (2006). F-box proteins everywhere. Curr. Opin. Plant Biol..

[60] Kipreos E.T., Pagano M. (2000). The F-box protein family. Genome Biol..

[61] Craig K.L., Tyers M. (1999). The F-box: A new motif for ubiquitin dependent proteolysis in cell cycle regulation and signal transduction. Prog. Biophys. Mol. Biol..

[62] Skaar J.R., Pagan J.K., Pagano M. (2009). SnapShot: F Box Proteins I. Cell.

[63] Skaar J.R., D’Angiolella V., Pagan J.K., Pagano M. (2009). SnapShot: F Box Proteins II. Cell.

[64] Nguyen K.M., Busino L. (2020). The Biology of F-box Proteins: The SCF Family of E3 Ubiquitin Ligases. Adv. Exp. Med. Biol..

[65] Schulman B.A., Carrano A.C., Jeffrey P.D., Bowen Z., Kinnucan E.R.E., Finnin M., Elledge S.J., Harper J., Pagano M., Pavletich N.P. (2000). Insights into SCF ubiquitin ligases from the structure of the Skp1–Skp2 complex. Nature.

[66] Mercer A.A., Fleming S.B., Ueda N. (2005). F-Box-Like Domains are Present in Most Poxvirus Ankyrin Repeat Proteins. Virus Genes.

[67] Sonnberg S., Fleming S.B., Mercer A. (2009). A truncated two-α-helix F-box present in poxvirus ankyrin-repeat proteins is sufficient for binding the SCF1 ubiquitin ligase complex. J. Gen. Virol..

[68] Sonnberg S., Seet B.T., Pawson T., Fleming S.B., Mercer A.A. (2008). Poxvirus ankyrin repeat proteins are a unique class of F-box proteins that associate with cellular SCF1 ubiquitin ligase complexes. Proc. Natl. Acad. Sci. USA.

[69] van Buuren N., Couturier B., Xiong Y., Barry M. (2008). Ectromelia Virus Encodes a Novel Family of F-Box Proteins That Interact with the SCF Complex. J. Virol..

[70] Werden S.J., Lanchbury J., Shattuck D., Neff C., Dufford M., McFadden G. (2009). The Myxoma Virus M-T5 Ankyrin Repeat Host Range Protein Is a Novel Adaptor That Coordinately Links the Cellular Signaling Pathways Mediated by Akt and Skp1 in Virus-Infected Cells. J. Virol..

[71] Price C.T., Al-Quadan T., Santic M., Jones S.C., Abu Kwaik Y. (2010). Exploitation of conserved eukaryotic host cell farnesylation machinery by an F-box effector of Legionella pneumophila. J. Exp. Med..

[72] Min C.-K., Kwon Y.-J., Ha N.-Y., Cho B.-A., Kim J.-M., Kwon E.-K., Kim Y.-S., Choi M.-S., Kim I.-S., Cho N.-H. (2014). Multiple Orientia tsutsugamushi Ankyrin Repeat Proteins Interact with SCF1 Ubiquitin Ligase Complex and Eukaryotic Elongation Factor 1 α. PLoS ONE.

[73] Werren J.H., Richards S., Desjardins C.A., Niehuis O., Gadau J., Colbourne J.K., Beukeboom L.W., Desplan C., Elsik C.G., Grimmelikhuijzen C.J. (2010). Functional and evolutionary insights from the genomes of three parasitoid Nasonia species. Science.

[74] Odon V., Georgana I., Holley J., Morata J., de Motes C.M. (2018). Novel Class of Viral Ankyrin Proteins Targeting the Host E3 Ubiquitin Ligase Cullin-2. J. Virol..

[75] Burles K., Irwin C.R., Burton R.-L., Schriewer J., Evans D.H., Buller R.M., Barry M. (2014). Initial characterization of Vaccinia Virus B4 suggests a role in virus spread. Virology.

[76] Mohamed M.R., Rahman M.M., Lanchbury J.S., Shattuck D., Neff C., Dufford M., van Buuren N., Fagan K., Barry M., Smith S. (2009). Proteomic screening of variola virus reveals a unique NF-κB inhibitor that is highly conserved among pathogenic orthopoxviruses. Proc. Natl. Acad. Sci. USA.

[77] Shchelkunov S., Safronov P.F., Totmenin A.V., Petrov N.A., Ryazankina O.I., Gutorov V.V., Kotwal G.J. (1998). The Genomic Sequence Analysis of the Left and Right Species-Specific Terminal Region of a Cowpox Virus Strain Reveals Unique Sequences and a Cluster of Intact ORFs for Immunomodulatory and Host Range Proteins. Virology.

[78] Ramsey-Ewing A.L., Moss B. (1996). Complementation of a Vaccinia Virus Host-Range K1L Gene Deletion by the Nonhomologous CP77 Gene. Virology.

[79] Perkus M.E., Goebel S.J., Davis S.W., Johnson G.P., Limbach K., Norton E.K., Paoletti E. (1990). Vaccinia virus host range genes. Virology.

[80] Mossman K., Lee S.F., Barry M., Boshkov L., McFadden G. (1996). Disruption of M-T5, a novel myxoma virus gene member of poxvirus host range superfamily, results in dramatic attenuation of myxomatosis in infected European rabbits. J. Virol..

[81] Needleman S.B., Wunsch C.D. (1970). A general method applicable to the search for similarities in the amino acid sequence of two proteins. J. Mol. Biol..

[82] Schoggins J.W. (2019). Interferon-Stimulated Genes: What Do They All Do?. Annu. Rev. Virol..

[83] Sarkar S.N., Sen G.C. (2004). Novel functions of proteins encoded by viral stress-inducible genes. Pharmacol. Ther..

[84] Pichlmair A., Lassnig C., Eberle C.-A., Górna M., Baumann C.L., Burkard T., Buerckstuemmer T., Stefanovic A., Krieger S., Bennett K.L. (2011). IFIT1 is an antiviral protein that recognizes 5′-triphosphate RNA. Nat. Immunol..

[85] Habjan M., Hubel P., Lacerda L., Benda C., Holze C., Eberl C.H., Mann A., Kindler E., Gil-Cruz C., Ziebuhr J. (2013). Sequestration by IFIT1 Impairs Translation of 2′O-unmethylated Capped RNA. PLoS Pathog..

[86] Daffis S., Szretter K.J., Schriewer J., Li J., Youn S., Errett J., Lin T.-Y., Schneller S., Zust R., Dong H. (2010). 2′-O methylation of the viral mRNA cap evades host restriction by IFIT family members. Nature.

[87] Verdonck S., Nemegeer J., Vandenabeele P., Maelfait J. (2022). Viral manipulation of host cell necroptosis and pyroptosis. Trends Microbiol..

[88] Dhuriya Y.K., Sharma D. (2018). Necroptosis: A regulated inflammatory mode of cell death. J. Neuroinflamm..

[89] Cho Y.S., Challa S., Moquin D., Genga R., Ray T.D., Guildford M., Chan F.K.-M. (2009). Phosphorylation-Driven Assembly of the RIP1-RIP3 Complex Regulates Programmed Necrosis and Virus-Induced Inflammation. Cell.

[90] Mohamed M.R., Rahman M.M., Rice A., Moyer R.W., Werden S.J., McFadden G. (2009). Cowpox Virus Expresses a Novel Ankyrin Repeat NF-kappaB Inhibitor That Controls Inflammatory Cell Influx into Virus-Infected Tissues and Is Critical for Virus Pathogenesis. J. Virol..

[91] Zhang Q., Lenardo M.J., Baltimore D. (2017). 30 Years of NF-κB: A Blossoming of Relevance to Human Pathobiology. Cell.

[92] Kerr P.J. (2012). Myxomatosis in Australia and Europe: A model for emerging infectious diseases. Antivir. Res..

[93] Johnston J.B., Wang G., Barrett J.W., Nazarian S.H., Colwill K., Moran M., McFadden G. (2005). Myxoma Virus M-T5 Protects Infected Cells from the Stress of Cell Cycle Arrest through Its Interaction with Host Cell Cullin-1. J. Virol..

[94] Tsvetkov L.M., Yeh K.-H., Lee S.-J., Sun H., Zhang H. (1999). p27Kip1 ubiquitination and degradation is regulated by the SCFSkp2 complex through phosphorylated Thr187 in p27. Curr. Biol..

[95] Wang G., Barrett J.W., Stanford M., Werden S.J., Johnston J.B., Gao X., Sun M., Cheng J.Q., McFadden G. (2006). Infection of human cancer cells with myxoma virus requires Akt activation via interaction with a viral ankyrin-repeat host range factor. Proc. Natl. Acad. Sci. USA.

[96] Werden S.J., Barrett J.W., Wang G., Stanford M., McFadden G. (2007). M-T5, the Ankyrin Repeat, Host Range Protein of Myxoma Virus, Activates Akt and Can Be Functionally Replaced by Cellular PIKE-A. J. Virol..

[97] Manning B.D., Toker A. (2017). AKT/PKB Signaling: Navigating the Network. Cell.

[98] Alessi D.R., Andjelkovic M., Caudwell B., Cron P., Morrice N., Cohen P., Hemmings B.A. (1996). Mechanism of activation of protein kinase B by insulin and IGF-1. EMBO J..

[99] Alessi D.R., James S.R., Downes C., Holmes A.B., Gaffney P.R., Reese C.B., Cohen P. (1997). Characterization of a 3-phosphoinositide-dependent protein kinase which phosphorylates and activates protein kinase Bα. Curr. Biol..

[100] Sarbassov D.D., Guertin D.A., Ali S.M., Sabatini D.M. (2005). Phosphorylation and Regulation of Akt/PKB by the Rictor-mTOR Complex. Science.

[101] Baffi T.R., Lordén G., Wozniak J.M., Feichtner A., Yeung W., Kornev A.P., King C.C., Del Rio J.C., Limaye A.J., Bogomolovas J. (2021). mTORC2 controls the activity of PKC and Akt by phosphorylating a conserved TOR interaction motif. Sci. Signal..

[102] Werden S.J., McFadden G. (2010). Pharmacological Manipulation of the Akt Signaling Pathway Regulates Myxoma Virus Replication and Tropism in Human Cancer Cells. J. Virol..

[103] Fujita N., Sato S., Katayama K., Tsuruo T. (2002). Akt-dependent Phosphorylation of p27Kip1Promotes Binding to 14-3-3 and Cytoplasmic Localization. J. Biol. Chem..

[104] Chen D.-Y., Fabrizio J.-A., Wilkins S.E., Dave K.A., Gorman J.J., Gleadle J.M., Fleming S.B., Peet D.J., Mercer A.A. (2017). Ankyrin Repeat Proteins of Orf Virus Influence the Cellular Hypoxia Response Pathway. J. Virol..

[105] Rani S., Roy S., Singh M., Kaithwas G. (2022). Regulation of Transactivation at C-TAD Domain of HIF-1α by Factor-Inhibiting HIF-1α (FIH-1): A Potential Target for Therapeutic Intervention in Cancer. Oxidative Med. Cell. Longev..

[106] Lando D., Peet D.J., Gorman J.J., Whelan D.A., Whitelaw M.L., Bruick R.K. (2002). FIH-1 is an asparaginyl hydroxylase enzyme that regulates the transcriptional activity of hypoxia-inducible factor. Genes Dev..

[107] Cockman M.E., Webb J.D., Kramer H.B., Kessler B.M., Ratcliffe P.J. (2009). Proteomics-based Identification of Novel Factor Inhibiting Hypoxia-inducible Factor (FIH) Substrates Indicates Widespread Asparaginyl Hydroxylation of Ankyrin Repeat Domain-containing Proteins. Mol. Cell. Proteom..

[108] Volz A., Sutter G. (2017). Modified Vaccinia Virus Ankara: History, Value in Basic Research, and Current Perspectives for Vaccine Development. Adv. Virus Res..

[109] Sperling K.M., Schwantes A., Staib C., Schnierle B.S., Sutter G. (2009). The Orthopoxvirus 68-Kilodalton Ankyrin-Like Protein Is Essential for DNA Replication and Complete Gene Expression of Modified Vaccinia Virus Ankara in Nonpermissive Human and Murine Cells. J. Virol..

[110] Liu B., Panda D., Mendez-Rios J.D., Ganesan S., Wyatt L.S., Moss B. (2018). Identification of Poxvirus Genome Uncoating and DNA Replication Factors with Mutually Redundant Roles. J. Virol..

[111] Chang S.-J., Hsiao J.-C., Sonnberg S., Chiang C.-T., Yang M.-H., Tzou D.-L., Mercer A.A., Chang W. (2009). Poxvirus Host Range Protein CP77 Contains an F-Box-Like Domain That Is Necessary to Suppress NF-κB Activation by Tumor Necrosis Factor Alpha but Is Independent of Its Host Range Function. J. Virol..

[112] Van Buuren N., Burles K., Schriewer J., Mehta N., Parker S., Buller R.M., Barry M. (2014). EVM005: An Ectromelia-Encoded Protein with Dual Roles in NF-κB Inhibition and Virulence. PLOS Pathog..

[113] Burles K., Van Buuren N., Barry M. (2014). Ectromelia virus encodes a family of Ankyrin/F-box proteins that regulate NFκB. Virology.

